# Distribution, Transfer, and Health Risk of Organochlorine Pesticides in Soil and Water of the Huangshui River Basin

**DOI:** 10.3390/toxics11121024

**Published:** 2023-12-15

**Authors:** Ruyue Yu, Yang Zhou, Shengxian Xu, Jing Jing, Hongyan Zhang, Yuanfang Huang

**Affiliations:** 1 College of Land Science and Technology, China Agricultural University, Beijing 100193, China; yry@cau.edu.cn (R.Y.);; 2 Innovation Center of Pesticide Research, Department of Applied Chemistry, College of Science, China Agricultural University, Beijing 100193, China

**Keywords:** distribution, source identification, level Ⅲ, health risks, Huangshui River Basin

## Abstract

The potential negative impacts of organochlorine pesticides on the environment and human health continue to receive attention. In order to study the spatial distribution characteristics of organochlorine pesticides in the inland alpine region, researchers collected soil and water samples in the Huangshui River Basin of the Qinghai–Tibetan Plateau and tested them for organochlorine pesticide residues represented by dichlorodiphenyltrichloroethane (DDT) and hexachlorohexane (HCH). The study identified the sources of OCPs by component analysis. We also constructed the LEVEL III model, applicable to the Huangshui River Basin, and used it to study the migration patterns of OCPs in various environmental media. OCPs were detected at low levels in the study area environment. The results of the OCPs source analysis show that there are both historical residuals and new sources in the region. Residual DDTs may originate from the mixture of technical DDTs and dicofol, and HCHs may originate from lindane or technical HCH. DDTs are mainly stored in soil, the input and output pathways are mainly atmospheric advection input and output, and its transport behavior in the environment is mainly air–soil exchange. Carcinogens in the study area pose little threat to people exposed to contaminated soil and contaminated water, but the cancer risk to children is greater than to adults. This study is helpful to managers of regional pesticide management and control.

## 1. Introduction

Organochlorine pesticides (OCPs) are a group of persistent organic pollutants (POPs) that were widely used in Asian countries until the 1980s because of their broad-spectrum insecticidal properties, their powerful and long-lasting efficacy, and their ease of production [1]. However, OCPs are highly persistent and toxic, have a low solubility in water, and are difficult to degrade in the environment, so they are of great concern to the international community and are gradually being banned. Dichlorodiphenyltrichloroethane (DDTs) and hexachlorocyclohexane (HCHs) were the earliest and most widely used OCPs, with China accounting for 33% (4.9 million tons) of global production of HCHs and 20% (400,000 tons) of global production of DDTs before production was banned in May 1983 [2]. Although OCPs have been banned, they can be transported even to remote polar and high altitudes regions with cold climates due to their long-range atmospheric transport and ‘cold condensation effect’ [3]. OCPs have been shown to be present in the Tibetan Plateau region [4,5].

Organic pollution at high altitudes can pose a threat to ecosystems [5]. Although many studies have focused on the distribution of OCPs, some major areas, such as the Tibetan Plateau, have received little attention [6]. Most of the studies on OCPs in the Tibetan Plateau have been conducted on the atmosphere and water, but there are few studies on OCPs in soils, especially agricultural soils. Meanwhile, many studies have shown that the largest sink for pesticides in the environment is probably soil, from which OCPs are released and migrate to the other components of the environment [7]. The Huangshui River Basin is located in Qinghai province, in the eastern part of the Qinghai–Tibet Plateau, which is an important area for protecting the ecological security of the Qinghai–Tibet Plateau, and it is highly sensitive to environmental changes [8]. More than 50% of Qinghai Province’s arable land is concentrated in the Huangshui River Basin, and about 69% of the province’s pesticides are used in the Huangshui River Basin [9]. It was found that OCPs posed a risk to both vegetables and humans within the Huangshui River Basin [10]. Therefore, it is necessary to study the contamination characteristics of OCPs in soils of typical areas of the Tibetan Plateau and their fate in various phases of the environment, which will provide a favorable theoretical basis for the protection of the ecological barrier on the Tibetan Plateau. Existing monitoring data are very limited, and many studies have utilized fugacity modeling to provide a holistic description of the fate and transport processes of OCPs in a variety of environmental media, including air, water, soil, and sediment. For example, Dong used the model to simulate the transfer and fate of HCHs since the 1950s in Lanzhou, China. Tasdemir studied the seasonal variations in levels and fluxes of air–soil exchange of PCBs in Turkey [11,12].

In this study, HCHs and DDTs (and their metabolites and isomers), which were once commonly used in the Huangshui River Basin, were taken as the research objects. Soil and water samples from the Huangshui River Basin were sampled and tested for analysis. The objectives were (1) to determine the concentrations and distribution characteristics of OCPs in water and soil samples; (2) to investigate the potential sources of OCPs; (3) to develop a fugacity model applicable to the Huangshui River Basin and to investigate the transport pathways of OCPs between the atmosphere–soil–water–sediment; and (4) to assess the health risks of OCPs in soil and water to adults and children. This study can provide a reference for the management and control of regional pesticides in highland areas.

## 2. Materials and Methods

### 2.1. Samples Collection

**Figure 1:**
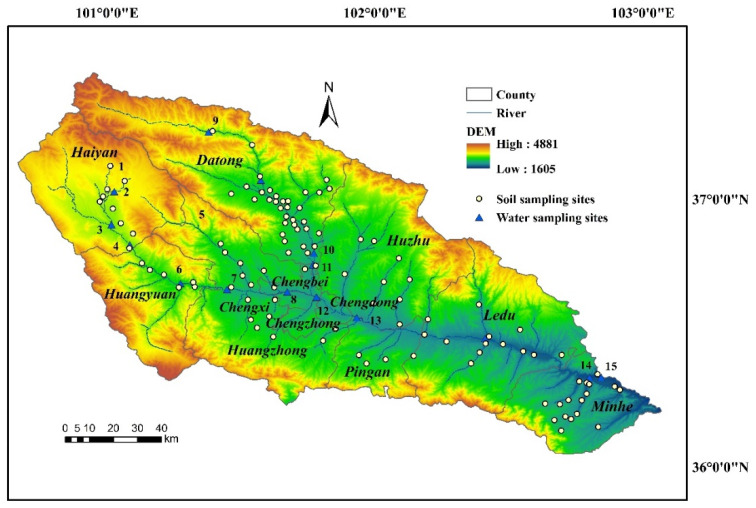
Location of sampling sites in the Huangshui River Basin. These numbers refer to water sampling points.

The Huangshui River is a major tributary of the upper reaches of the Yellow River, flowing from west to east through 12 districts and counties. The Huangshui River Basin (36°02′–37°28′ N, 100°41′–103°04′ E) is located in the eastern agricultural region of Qinghai Province. The main land use types are classified as farmland, grassland, and woodland, and the main soil types are calcareous soil, chernozem, chestnut soil, and meadow soil. The location of the sites took into account the soil type, land-use type, vegetation cultivation type, clay content, and location of point source pollution in the study area. A total of 110 soil samples and 15 water samples were eventually collected in April 2021 from the upper, middle, and lower Huangshui River Basin and typical tributary areas (Figure 1).

Using the five-point sampling method, a sample was taken at a depth of 0–20 cm and 1.0 kg was collected at each point using a stainless steel spatula. After mixing well, the sample was retained at around 2.5 kg according to the quartering method. The collected soil was placed in a self-sealing bag and sent back to the laboratory for cryopreservation and analysis.

The water sample points were mainly selected from the monitoring sections of the state-controlled rivers and the provincial-controlled rivers, and 15 water sample points were arranged at the estuary of the mainstream and tributaries (Figure 1). Water samples were collected in strict accordance with the “Water quality sampling—technical regulation of the preservation and handling of samples (HJ 493—2009)”, and water samples were immediately put into the insulation box filled with ice packs, and transported back to the laboratory in the 4 °C conditions to be preserved for testing.

### 2.2. Chemicals

All samples were analyzed for OCPs, including DDTs (*p,p′*-DDE, *p,p′*-DDD, *o,p′*-DDT, and *p,p′*-DDT) and HCHs (α-HCH, β-HCH, γ-HCH, and δ-HCH). Pesticide standards were purchased from ANPEL Laboratory Technologies (Shanghai, China) Inc. Octadecyl-silica (C18) was obtained from Agela Technologies, Tianjin, China. Reaction-grade formic acid, sodium chloride (NaCl), and magnesium sulfate anhydrous (MgSO_4_) were purchased from the Beijing Chemical Reagent Company (Beijing, China). HPLC-grade n-hexane, acetone, acetonitrile, and methanol were provided by ANPEL Laboratory Technologies (Shanghai) Inc. (Shanghai, China), too. The ultrapure water was prepared by the Aquapro Ultrapure Water System. The DDTs and HCHs were prepared in a 100 mg L^−1^ mixed standard solution with n-hexane.

### 2.3. Extraction and Analysis

For the determination of HCHs and DDTs in soil, the modified method described by Zhang was adopted in the extraction program [13].

The pretreatment process of DDTs and HCHs in water is as follows: the uniformly mixed 20 mL water sample was added with 20 mL hexane, vortexed at 3800 rpm for 5 min, centrifuged for 5 min, and all the supernatant was transferred to a chicken heart bottle. The water bath was evaporated to nearly dry in a 45 °C water bath, and 1 mL hexane was taken for constant volume. The stopper was covered during the constant volume process, and all the solutions were absorbed using a disposable syringe, filtered through the membrane for analysis.

### 2.4. Method Validation

To validate the accuracy of the method, three levels, namely, 0.005, 0.01, and 0.1 mg kg^−1^ were verified for DDTs and HCHs based on GC–ECD, in triplicate. The recoveries of pesticides in soil ranged from 72% to 105%. The linearity was evaluated using matrix matching calibration curves for HCHs and DDTs by GC–ECD. The matrix external standard method was used to determine the pesticides in unknown samples. The results were highly linear with R^2^ > 0.99 in the concentration range from 0.001 to 0.1 mg kg^−1^ for GC–ECD analysis of DDTs and HCHs. All conditions met the requirements for analysis of OCPs in subsequent samples. The limit of quantification (LOQ) for each pesticide was estimated as the concentration at a signal-to-noise ratio (S/N) of 10. Concentrations below the LOQ were considered non-detected and are indicated by “ND”. Pesticide residue concentrations ≥ LOQ were considered in the data analysis, while concentrations < LOQ were not analyzed [10]. The detailed data of quality control are listed in Table S1

### 2.5. OCPs Transport Modeling

This study applies the LEVELIII fugacity model developed by Canadian scholars to solve the model (http://www.trentu.ca/, accessed on 1 July 2023). Because only *p,p′*-DDE was detected in the soil, to ensure the accuracy of the results, this part of the study was conducted only with *p,p′*-DDE. The framework and equations of the model are similar to those used in a previous study for OCPs in Lanzhou, China and a gas–soil exchange study for PCBs in Turkey [12,14]. In this paper, the Huangshui River Basin is regarded as a system, in which the atmosphere, water, soil, and sediment are selected as four main environmental phases, and the transport processes involved include diffusion between the atmosphere and water and soil, rainwater dissolution, dry and wet deposition, soil runoff, sediment resuspension, and other processes. Using the LEVEL III model, a three-stage, steady-state, multi-media transport normalization model of *p,p′*-DDE was developed for watersheds to analyze the migration patterns of *p,p′*-DDE in multi-media environments under steady-state, non-equilibrium, and systematic flow conditions.

The fugitive capacity of air, soil, water, and sediment can be estimated by the equation, and the equilibrium partition coefficient between each environmental phase enables the estimation of the value of the fugitive capacity of pollutants in other environmental phases. The mass balance equations for each phase are as follows:

(1) $$E_{1}+G_{A1}c_{B1}+f_{2}D_{21}+f_{3}D_{31}=f_{1}(D_{12}+D_{13}+D_{R1}+D_{A1})$$

(2) $$E_{2}+G_{A2}c_{B2}+f_{1}D_{12}+f_{3}D_{32}+f_{4}D_{42}=f_{2}(D_{21}+D_{24}+D_{R2}+D_{A2})$$

(3) $$E_{3}+f_{2}D_{23}=f_{3}(D_{32}+D_{R3}+D_{A3})$$

(4) $$E_{4}+f_{1}D_{14}=f_{4}(D_{41}+D_{42}+D_{R4})$$

where *E_i_* is the discharge rate (mol h^−1^); *G_A_* is the advective inflow rate (m^3^ h^−1^); *C_Bi_* is the advective inflow concentration [(mol m^3^)^−1^]; *D_Ri_* and *D_Ai_* denote the reaction rate and advective rate *D* values, respectively; and *D_n_* denotes the sum of all media *i* loss *D* values.

A total of 75 parameters were used in this study, including parameters of environmental characteristics of the Huangshui River Basin, physicochemical properties of *p,p′*-DDE, and migration parameters of polluted *p,p′*-DDE. Among them, because the average temperature of Huangshui River Basin is low, according to the method provided by Paasivirta [15], we transformed the physicochemical properties of *p,p′*-DDE under 25 °C to those under 4 °C (the average temperature of the Huangshui River Basin). All parameters were collected from the literature or measured in the laboratory. The specific selection parameters of the model are shown in Table S2.

### 2.6. Health Risk Assessment

In general, individuals are exposed to OCPs in the soil through three pathways: direct ingestion, dermal absorption, and inhalation [16,17]. The equations and parameters of Ma and Hu were applied in this study [17,18]. The average daily doses (ADD, mg kg^−1^ per day) of the 3 ways were calculated using the following equations [19,20]:

(5) $$\mathrm{ADD}\mathrm{ing}=\frac{Cs\times \mathrm{IR}\mathrm{ing}\times \mathrm{EF}\times \mathrm{ED}}{\mathrm{BW}\times \mathrm{AT}}\times \mathrm{CF}$$

(6) $$\mathrm{ADD}\mathrm{der}=\frac{\mathrm{CS}\times \mathrm{SA}\times \mathrm{AF}\times \mathrm{ABS}\times \mathrm{EF}\times \mathrm{ED}}{\mathrm{BW}\times \mathrm{AT}}\times \mathrm{CF}$$

(7) $$\mathrm{ADD}\mathrm{inh}=\frac{\mathrm{CS}\times \mathrm{IR}\mathrm{inh}\times \mathrm{EF}\times \mathrm{ED}}{\mathrm{PEF}\times \mathrm{BW}\times \mathrm{AT}}$$

where ADD_ing_, ADD_inh,_ and ADD_der_ are calculated ADD from ingestion, inhalation, and dermal routes, respectively. Cs represents the concentration of the target pollutants in soil (mg kg^−1^). In this study, the maximum value of Cs was selected for the calculation of the ADDs. IR_ing_ denotes the intake rate (mg per day), EF is the exposure frequency (day per year), ED is the exposure duration (year), CF is the conversion factor (kg mg^−1^), BW represents the body weight (kg), AT is the average exposure time (day), SA is the skin exposure area that contacts the soil (cm^2^ per day), AF is the skin adherence factor(mg per cm^2^), ABS is the dermal absorption factor (unitless), IR_inh_ is the inhalation rate(m^3^ per day), and PEF is the particle emission factor (m^3^ kg^−1^); carcinogenic risk (CR_soil_) in the soil is calculated using the following formula:

(8) CRsoil=ADD×SFs

where SF_s_ is carcinogenic slope factor [(mg kg^−1^ per day)^−1^] [17].

The chronic daily intake (CDI) is used to assess human exposure to contaminants via the oral exposure route [21], and is calculated by the following equation,

(9) $$\mathrm{CDI}=\frac{\mathrm{Cw}\times \mathrm{IR}\times \mathrm{EF}\times \mathrm{ED}}{\mathrm{BW}\times \mathrm{AT}}$$

C_w_ = chemical concentration in water (mg L^−1^); IR = water ingestion rate (L per day), carcinogenic risk (CR _water_) in water is calculated as follows:

(10) CRwater=CDI×SFw

CDI is the chronic daily intake from drinking water (mg kg^−1^ per day), and SF_w_ is the slope factor of the pollutant via exposure route [(mg kg^−1^ per day)^−1^].

The U.S. EPA has defined the excess lifetime carcinogenicity target risk level for carcinogens in soil as less than one in one million (1 × 10^−6^). Therefore, in the carcinogenic risk assessment, values below 1 × 10^−6^ (one case per million exposed people) is considered as a negligible risk, a value between 10^−6^ and 10^−4^ was low, a value between 10^−4^ and 10^−3^ was moderate, and a value higher than 10^−1^ was high. The specific parameters are shown in Tables S3 and S4, and the parameters are taken from the website (https://www.epa.gov/iris, accessed on 1 July 2023) and Li [22].

### 2.7. Data Analysis

The software Excel 2010 (Microsoft Inc., Seattle, WA, USA) was used for statistical data analysis, and Origin 2019b (Origin Lab Inc., Northampton, MA, USA) and ArcGIS 10.4 (Environmental Systems Research Institute Inc., Redlands, CA, USA) were used for chart and map drawing, respectively.

## 3. Result and Discussion

### 3.1. Occurrence of OCPs in Soil and Water Samples

Of all metabolites and isomers of HCHs and DDTs, only *p,p′*-DDE was detected in 35 soil samples, with a frequency of 31.8%. The maximum value of 19 μg kg^−1^ for *p,p′*-DDE in the whole basin occurred in Minhe County. The *p,p′*-DDE does not follow a normal distribution in the basin and has a large degree of variation, with a coefficient of variation of 212%. The high CVs indicate that the spatial distribution of pesticides is strongly interfered with by human activities. According to the agricultural soil pollution risk screening values (100 μg kg^−1^) in China (GB 15618-2018), soils in the Huangshui River Basin are not contaminated with OCPs.

Similar to the study area, HCHs were not detected in soils near the Himalayas, which are also on the Tibetan Plateau, and DDTs were only present at levels of 0.39–6.06 ng g^−1^ [5]. In contrast, HCHs were detected in the surface sediments of Qinghai Lake at concentrations of 0.02–1.00 ng g^−1^ and DDTs at a maximum of 0.86 ng g^−1^, slightly lower than in the Huangshui River Basin [4]. The residues of organochlorine pesticides in the soils of the Alps (0.4–28.8 μg kg^−1^ for *p,p′*-DDT, 0.3–8.8 μg kg^−1^ for γ-HCH), also an alpine region, were higher than in the Huangshui River Basin [3]. The mean concentrations of DDTs in the soils of Binhai New Area of Tianjin reached 73.9 μg kg^−1^ and HCHs reached 666 μg kg^−1^; the concentrations of DDTs in the soils of the estuary of Bohai Bay ranged from 98.32–129.10 μg kg^−1^ and HCHs ranged from 69.81–379.28 μg kg^−1^, both much higher than concentrations in the Huangshui River Basin. The Huangshui River Basin is far from areas where OCPs are used in large quantities, and they have much lower residue levels than those in areas of high agricultural activity.

The detection frequency and mean of *p,p′*-DDE in each district and county are shown in Table 1, where Chengzhong, Chengxi, Chengdong, and Chengbei districts are combined as urban areas. To determine the level of contamination in each district and county in the basin, we calculated the mean and detection frequency of *p,p′*-DDE residues in each district and county to compare them to those of the total sample points. If the mean and frequency of a district or county are both lower than that of the total sample point, it is labeled as green; if they are both higher, it is labeled as red; and the rest is labeled as yellow.

**Figure 2:**
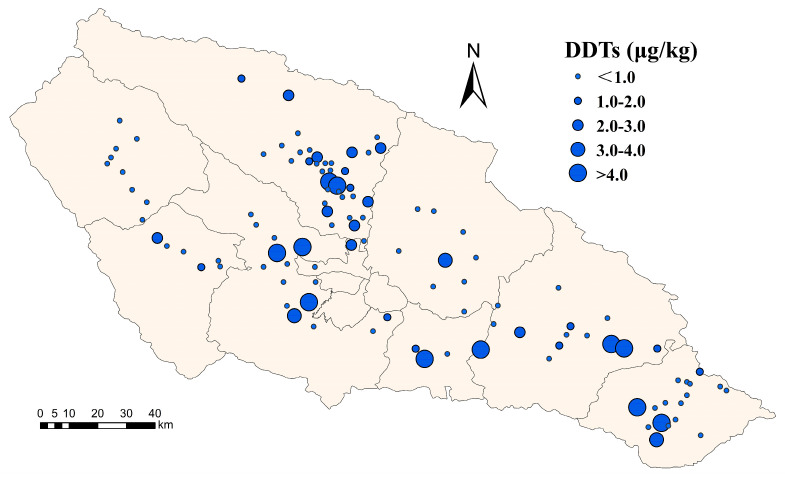
Spatial distribution of *p,p′*-DDE in the Huangshui River Basin.

Spatially, organochlorine pesticides are sporadically distributed throughout the watershed, with small aggregations in the middle and lower reaches of the basin, and low levels of detection in the upper reaches (Table 1 and Figure 2). From the 1940s to the 1970s, organochlorine pesticides were the predominant pesticides in the study area [23]. Despite control measures, *p,p′*-DDEs are still detected throughout the basin because of their long-range transport and environmental persistence [24]. In general, the detection level of *p,p′*-DDE in the upper part of the watershed is lower than that in the lower part of the watershed. *p,p′*-DDE was not detected in Haiyan County in the northwestern part of the study area, which is located in the upper part of the Huangshui Basin with high elevation, low temperature, limited agricultural activities, and a late sowing period. Locations with high *p,p′*-DDE residue levels were mainly concentrated in the middle and lower reaches of the basin. Higher detection levels of *p,p′*-DDE in downstream watersheds such as Ledu and Ping’an Districts may be due to the pooling of pesticide residues from the upstream and midstream watersheds into the lower reaches of the river, in addition to the high level of agricultural activities of the population in the area.

OCPs were detected in some water bodies of the Huangshui River (Table 2). Unlike in soil (HCHs were not detected), DDTs and HCHs were both detected in water samples. Different physicochemical properties of HCHs and DDTs may be partially responsible for this phenomenon. Compared with DDTs, HCHs are less lipophilic and more soluble in water and may be transported to rivers more easily and quickly. In the meantime, DDTs are more stable than HCHs, and they degrade slowly in soil. Therefore, although HCHs were not detected in soil, the concentration levels of HCHs in water were close to that of DDTs [4]. In comparison, OCPs had higher detection frequency in the upstream and downstream of the basin, and almost no detection in the midstream.

### 3.2. The Sources and Compositions of OCPs

OCPs have been banned for many years, yet small amounts were still detected. Therefore, it is important to investigate the source of OCPs and whether there is any new contamination input from the recent years.

#### 3.2.1. The Sources and Compositions of DDTs

In the natural environment, DDTs will gradually biodegrade to *p,p′*-DDE and *p,p′*-DDD. The longer DDTs exist in the environment, the higher the proportion of degradation products is, and the proportion of DDT is relatively lower. Therefore, the ratio of DDT/(DDE + DDD) can be used to determine whether DDTs are fresh pollutant. DDT/(DDE + DDD) < 1 is indicative of long-term weathering (microbially degraded) of DDTs, and DDT/(DDE + DDD) > 1 indicates fresh application or new sources [1]. In this study, *p,p′*-DDD and *p,p′*-DDT were not detected in the soil, which indicates that all the DDTs in the soil was degraded and no new sources of DDTs had been inputted in to the soil. DDT/(DDE + DDD) was less than 1 in most of the water samples, with only two point ratios of 1.08 and 1.23, respectively. This indicates that the DDTs in most of the water sample sites have been completely decomposed, but there are still some sites that may have fresh DDTs input.

**Figure 3:**
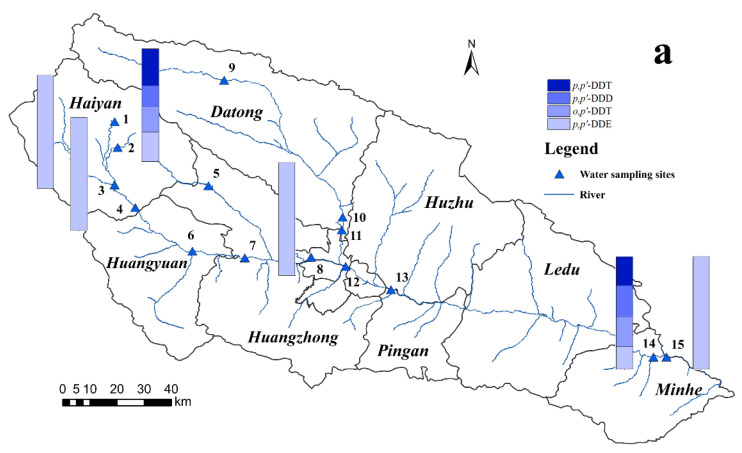

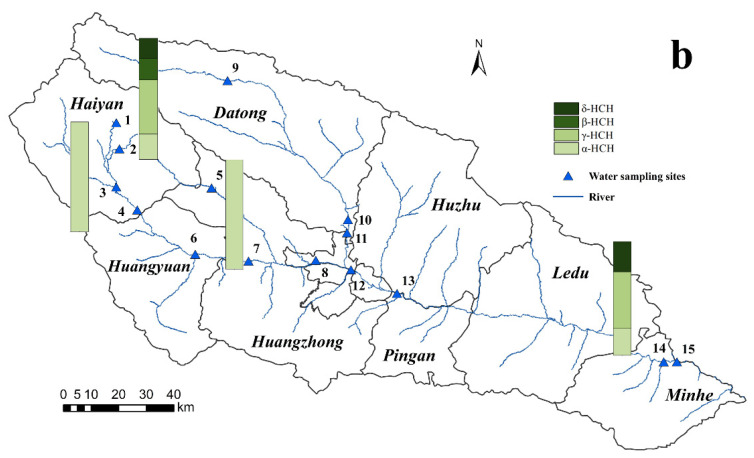
Spatial distributions of DDTs (**a**) and HCHs (**b**) in the water of the Huangshui River Basin. These numbers refer to water sampling points.

Sources of DDTs include both technical DDT and dicofol. After technical DDT was banned as a pesticide in China in the 1980s, dicofol was used as an insecticide for crops such as cotton, fruit trees, and tea trees [25]. Technical DDT is still used as an additive in the production of antifouling paints for fishing boats [26]. Generally, technical DDT contains 75% *p,p′*-DDT, 15% *o,p′-*DDT, 5% *p,p′-*DDE, and 5% of other components, with *o,p′*-DDT/*p,p′*-DDT ratios ranging from 0.2 to 0.3 [1,27]. In dicofol, *o,p′*-DDT is more abundant than *p,p′-*DDT, and the ratio of *o,p′*-DDT/*p,p′*-DDT is about 7.5. For this reason the ratios of *o,p′*-DDT/*p,p′*-DDT are often used for estimating the sources of DDTs [28]. In this study, *o,p′*-DDT and *p,p′*-DDT were not detected in any soil samples (Figure 3a). The residuals of *o,p′*-DDT and *p,p′*-DDT were only detected in samples 2 and 14. The ratios of *o,p′*-DDT/*p,p′*-DDT at these two sites were 0.69 and 1.03, which were higher than the content in the technical DDT mixture, but much lower than 7.5, indicating that DDTs at these two sites might originate from the mixture of early technical DDT and dicofol, and technical DDT was dominant.

#### 3.2.2. The Sources and Compositions of HCHs

In the environment, α-HCH and γ-HCH are relatively more volatile and readily lost in sediments; α-HCH and γ-HCH could be converted to β-HCH in aged environments. β-HCH is more resistant to hydrolysis and biodegradation, and is the most stable isomer. A high ratio of β-HCH/(α-HCH + γ-HCH) indicates that HCHs are mainly historical pollution [29]. Otherwise, it indicates that HCHs originate from the recent use of pesticides or the dry and wet deposition of the atmosphere [30]. The ratio of β-HCH/(α-HCH + γ-HCH) at site 2 was 0.97, but the residues of β-HCH in sites 4, 7, and 14 were lower than the detection limit. This indicates that there are both historical residuals and other sources of HCHs in the region, such as through atmospheric drift and dry and wet deposition.

HCH products can generally be divided into two major types: technical HCH and lindane. After 1991, technical HCH used in China was replaced by lindane, which is still used there [31]. Generally, technical HCH contains 55–80%, 5–14%, 8–15%, and 2–16% α-HCH, β-HCH, γ-HCH, and δ-HCH, respectively. The ratio of α-HCH/γ-HCH in technical HCHs is between 4 and 7; the content of γ-HCH in lindane is greater than 99%, so the ratio of α-HCH/γ-HCH is usually low. Therefore, a high ratio of α-HCH/γ-HCH indicates that HCHs may come from technical HCHs; and HCHs with low ratios tend to come from lindane [27]. The source of HCHs in site 4 and site 7 was technical HCH due to the fact that the α-HCH percentage was highest (Figure 3b), and the source in site 2 and site 14 was mainly lindane because the ratio α-HCH/γ-HCH was low.

### 3.3. Environmental Transport of OCPs

**Figure 4:**
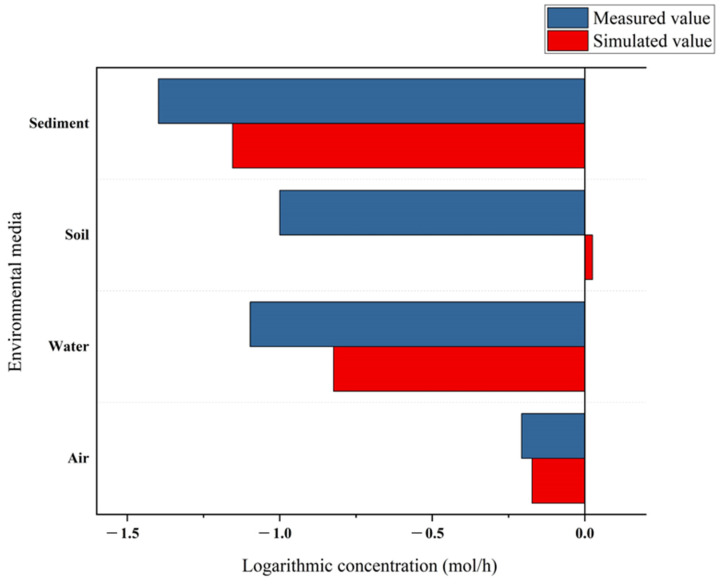
Correspondence between simulated and measured values.

The LEVEL III model was used to simulate the storage capacity of *p,p′*-DDE in each environmental phase of the Huangshui River Basin and its migration pattern between each environmental phase. The difference of about one order of magnitude between the estimated and measured values (log concentrations) of the model was considered reasonable, indicating that the model can provide a more objective description of the multi-media environmental behavior of organic pollutants in the study area [32]. Concentration of *p,p′*-DDE with fugacity obtained from the model simulation values and measured values in each environmental phase in the Huangshui River Basin are shown in Table 3 and Figure 4. It can be seen from the figure that the concentrations of the measured values are all higher than the concentrations of the model simulated values. The differences between modeled and actual measurements (log concentrations) of *p,p′*-DDE in the atmosphere [6,33], water, and sediments [4] are within an order of magnitude in the range of 0.03–0.3, while those in soil are of one order of magnitude. It shows that the model has a better consistency with the actual situation in simulating the distribution and convergence of *p,p′*-DDE in the Huangshui River Basin. Compared with the air, water, and sediment phases, the measured values in the soil phase are significantly higher than the simulated calculated values, probably due to the input of point sources of OCPs, which results in the measured values being generally larger than the simulated values.

**Figure 5:**
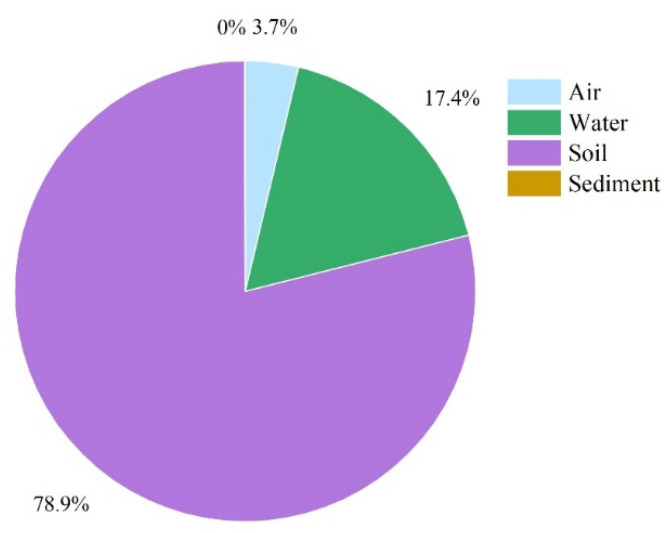
Storage of *p,p′*-DDE in various environmental phases in the Huangshui River Basin.

As shown in Figure 5, *p,p’*-DDE is most abundant in soil, and about 79% of *p,p′*-DDE eventually converges to soil. As the primary environmental phase for pesticide application, it offers a greater saving advantage over all other environmental phases. In addition, soil has no advective export effect and it adsorbs *p,p′*-DDE, so the accumulation there is likely to be greatest.

**Figure 6:**
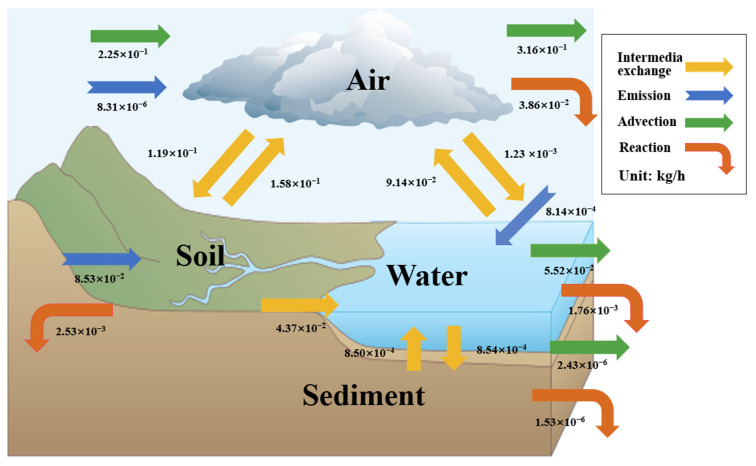
Main environmental behaviors of *p,p′*-DDE and their transport fluxes between different environmental media.

**Figure 7:**
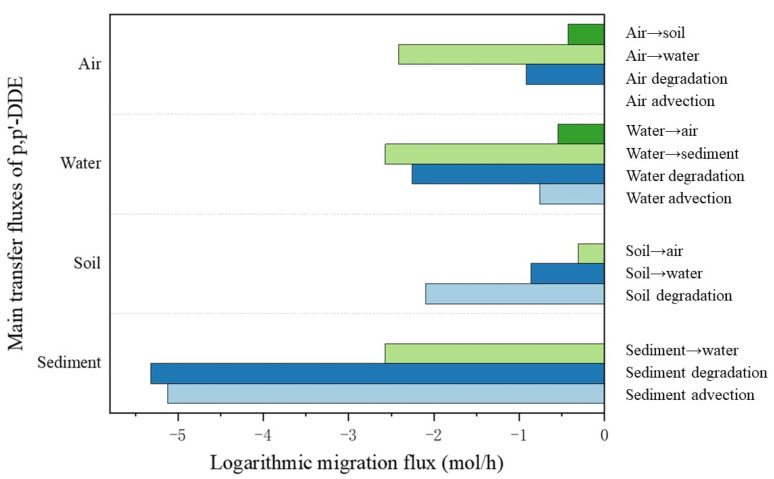
Major environmental behavioral fluxes of *p,p′*-DDE in various environmental media. The arrows between the media in the figure show the migration paths.

From the model results, we can see that when we consider the Huangshui River Basin as a whole environmental system, the transport of *p,p′*-DDE in the system is not unidirectional; it flows in each phase until the distribution and output of *p,p′*-DDE in the system in each environmental medium reaches steady state. The transport flux of organochlorine pesticides between environmental media in the model result is the transport flux when the system reaches steady state. As shown in Table 4, the primary pathway for the disappearance of *p,p′*-DDE from the study area was advective output. The main environmental behaviors of *p,p′*-DDE were manifested in the advective input and output of the atmosphere, and the transport between the atmosphere and soil. The Huangshui River Basin has arid areas and a dry climate, meaning rainfall measurements are small, so precipitation, rivers, and other pollutants carrying the role of migration is not obvious. At the same time, it has low vegetation cover, which is susceptible to sandstorms and dust. Thus, it is susceptible for organochlorine pollutants to be transported between the soil and the atmosphere (Figure 6 and Figure 7). In addition to inter-media transport, degradation losses are the main export pathway for *p,p′*-DDE in each environmental medium.

The main disappearance pathway of *p,p′*-DDE was atmospheric advection export, in the gas phase, 66.54% of *p,p′*-DDE was exported by atmospheric advection, and only 25.3% entered into the water body and soil through diffusion, rainwater dissolution, and wet and dry deposition. In the aqueous phase, about 37% of *p,p′*-DDE was exported by advection, and more than half of it entered into the atmosphere through volatilization, but only a very small portion of it was degraded or entered into the sediments through deposition. In the soil, about 77% entered the atmosphere through volatilization, while about 21% was transported out of the soil by surface runoff and other means. In the sedimentary phase, most of it entered the water column through diffusion, and a very small portion of it was no longer involved in the process of exchange between environmental phases.

Similar to the present study, the study results of Dong showed that POPs in Lanzhou were mainly stored in the soil phase (98.9%) and were mainly exported by atmospheric advection inputs [11]. However, the highest concentrations of organochlorine pesticides in Fujian Jinjiang Basin and Hainan Island were found in the sedimentary phase, the input source was mainly surface runoff input, and the disappearance pathway in the environment was mainly the degradation of environmental phases, especially the sedimentary phase [34]. It can be found that there are obvious differences in the transfer process between the Huangshui River Basin and these areas. Significant differences in the environment exist between coastal areas and the Huangshui River Basin. Coastal areas are affected by the oceanic climate, rainfall is sufficient, and river and ocean currents broadly distribute pollutants. In contrast, the Huangshui River Basin climate is arid, the precipitation and sedimentation rates are small, and the migration processes mainly occur between the atmosphere and soil. Secondly, the main source of *p,p′*-DDE in the Huangshui River Basin is pesticides applied to the soil in agricultural activities, while *p,p′*-DDE in the environment in the coastal area mainly comes from local agricultural uses and wastewater discharged from pesticide factories [14]. Therefore, attention needs to be paid to organochlorine pesticides in the soils of the study area.

### 3.4. Carcinogenic Risk Assessment of OCPs

As shown in the result of 3.1, the concentrations of DDTs and HCHs in soil are all below the standard value (100 μg kg^−1^) determined by the risk control standard for soil contamination. In addition, the content of DDTs and HCHs in almost all water sample is also lower than the limit standard for drinking water quality (5 µg L^−1^ for HCHs and 1 µg L^−1^ for DDTs). Because DDTs and HCHs are persistent organic pollutants with a carcinogenic effect, in addition to calculating their environmental risks, the calculation of carcinogenic risk to the human body is necessary [17].

**Figure 8:**
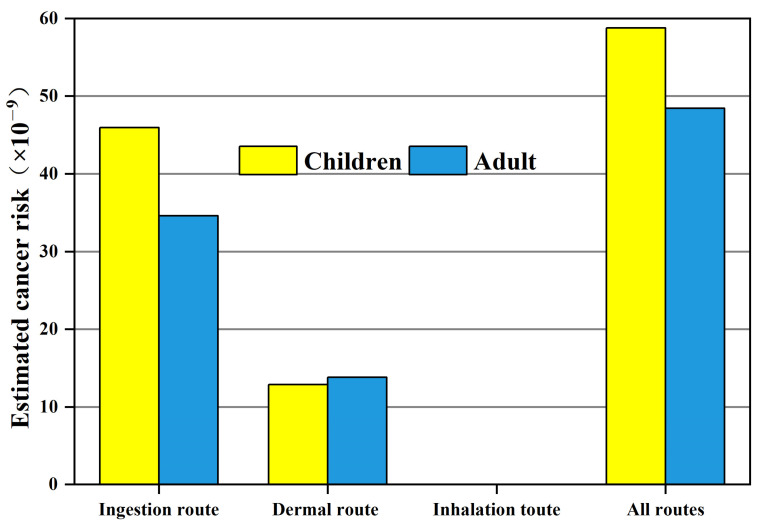
Estimated carcinogenic risk through various routes for children and adults.

Carcinogenic risk of OCPs in soil for children and adults in the Huangshui River Basin was calculated considering ingestion, inhalation, dermal routes, and total carcinogenic risk. Figure 8 lists the carcinogenic risk in the soil from the three ways, and they are all below 1 × 10^−6^, which indicates that carcinogenic risk is not a serious threat to the population exposed to contaminated soil [35]. The carcinogenic risks of OCPs were mainly derived from the ingestion route and dermal route. It is worth noting that OCPs pollutants pose a greater carcinogenic risk to children than adults. It is more common in children because they are likely to ingest soil inadvertently through hand-to-mouth activities while playing in the soil. Higher intake rates and lower body weights in children may result in higher doses of hazardous substances per unit of body weight [20]. Consistent with Ma’s study, the cancer risk in both adults and children from ingestion route was highest, with the trend of: ingestion > dermal contact > inhalation [17].

**Figure 9:**
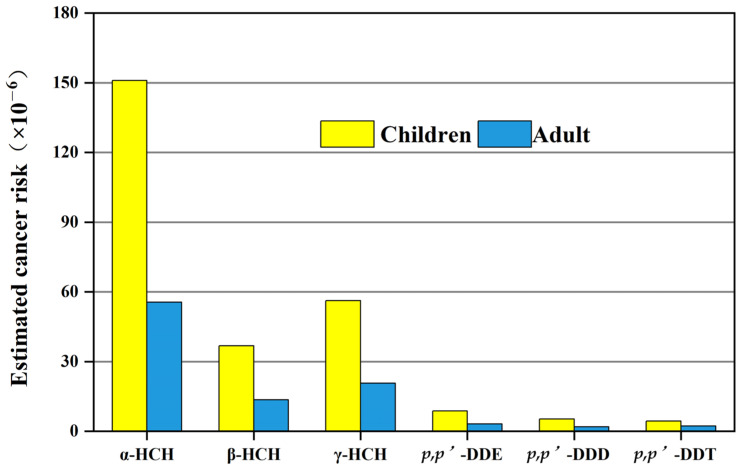
Estimated carcinogenic risk through water for children and adults.

Similar to the Yellow River estuary, the cancer risk of HCHs is higher than DDTs in the Huangshui River Basin, and the risk of α-HCH is the highest among the studied organochlorine pesticides [22]. For OCPs in water, the risk value fell in the range of 10^−8^ to 10^−4^, meaning carcinogenic risks were acceptable to both adults and children, but not low enough (Figure 9). Notably, the risk of cancer in children is more than twice that of adults. Consistent with other studies, children are more sensitive to the health risks of these pollutants [18]. Therefore, the local authorities should make their best efforts to reduce OCPs contamination in the environment, and should consider applying engineering measures to reduce the residual amount of OCPs and develop a circular economy if necessary [36].

## 4. Conclusions

This study reveals the pollution characteristics, sources, and migration patterns of DDTs and HCHs in the soil and water of the Huangshui River Basin, and assesses their health risks to human beings. Only *p,p′*-DDE is detected in the soil, which is lower than the level of detection of OCPs in soil in many parts of the world. Isomers and metabolites of DDTs and HCHs were detected in some water bodies. OCPs in soil and water bodies in the study area are generally at relatively clean levels. The composition analysis indicates that there are both historical residuals and new sources of OCPs in the region, such as from other areas entering the study area through atmospheric drift and deposition. DDTs in soil and water might originate from the mixture of technical DDTs and dicofol. The source of HCHs was mainly technical HCHs. DDTs are mainly stored in soil, the input and output pathways are mainly atmospheric advection input and output, and its transport behavior in the environment is mainly air–soil exchange. Carcinogens in the study area pose limited threat to people exposed to contaminated soil and contaminated water, but the cancer risk to children is greater than to adults in both cases.

## Figures and Tables

**Table 1:** The detection frequency and mean of *p,p′*-DDE in each district and county.

Location of Districts and Counties		Frequency (%)	Mean (μg kg^−1^)
Haiyan	Upstream	0.00	0
Huangyuan		33.30	0.77
Datong		37.10	0.99
Huzhu	Midstream	10.00	0.34
Urban areas		50.00	1.1
Huangzhong		33.30	1.71
Minhe	Downstream	23.50	1.81
Pingan		66.70	2.77
Ledu		53.80	1.95
Whole basin		31.80	1.22

Red: high risk value; yellow: medium risk value; green: low risk value.

**Table 2:** Summary of OCPs concentrations in water in the Huangshui River Basin.

	Frequency	Range	Mean
	(%)	(μg L^−1^)	(μg L^−1^)
α-HCH	26.67	ND-0.75	0.11
γ-HCH	13.33	ND-1.6	0.15
β-HCH	6.67	ND-0.64	0.04
δ-HCH	13.33	ND-0.59	0.06
*p,p′*-DDE	40	ND-0.8	0.15
*o,p′*-DDT	13.33	ND-0.69	0.07
*p,p′*-DDD	13.33	ND-0.57	0.06
*p,p′*-DDT	13.33	ND-1	0.09

**Table 3:** Comparison of LEVEL III model simulated *p,p′*-DDE values with actual measured values.

Environmental Phase	Measured Value	Simulated Value	Data Sources
Air	672 (pg m^3^)^−1^	621 (pg m^3^)^−1^	The literature
Water	0.15 μg L^−1^	0.08 μg L^−1^	Determination in the laboratory
Soil	1.06 μg kg^−1^	0.10 μg kg^−1^	Determination in the laboratory
Sediment	0.07 μg kg^−1^	0.04 μg kg^−1^	The literature

**Table 4:** The migration flux of *p,p′*-DDE in various environmental media (kg h^−1^).

Migration Flux	Transport Rate
Air to water	1.23 × 10^−3^
Air to soil	1.19 × 10^−1^
Water to air	9.14 × 10^−2^
Water to sediment	8.54 × 10^−4^
Soil to air	1.58 × 10^−1^
Soil to water	4.37 × 10^−2^
Sediment to water	8.50 × 10^−4^
Air advection	3.16 × 10^−1^
Water advection	5.52 × 10^−2^
Soil advection	-
Sediment advection	2.43 × 10^−6^
Air reaction	3.86 × 10^−2^
Water reaction	1.76 × 10^−3^
Soil reaction	2.53 × 10^−3^
Sediment reaction	1.53 × 10^−6^

## Data Availability

The original data presented in the study are included in the article/Supplementary Material; further inquiries can be directed to the corresponding author.

## Supplementary Materials

The following supporting information can be downloaded at: https://www.mdpi.com/article/10.3390/toxics11121024/s1, Table S1: Method validation results (Retention time, LOQ, calibration curves, R2, recoveries and RSD) of HCHs and DDTs with GC-ECD; Table S2: Parameters required to construct a fugacity model for the Huangshui River Basin; Table S3: SF and RfD values (Children) of the compounds detected in Huangshui River Basin; Table S4: SF and RfD values (Adult) of the compounds detected in Huangshui River Basin.
