# Supplementary material for: Distribution, Transfer, and Health Risk of Organochlorine Pesticides in Soil and Water of the Huangshui River Basin
Source: Toxics. 2023 Dec 15;11(12):1024. doi: 10.3390/toxics11121024 (PMC10747045; doi:10.3390/toxics11121024)
Supplement: Supplementary file 1 [file toxics-11-01024-s001.zip › toxics-2728620-supplementary.pdf]

Supplementary materials

Distribution, Transfer, and Health Risk of Organochlorine Pesticides in Soil and Water of the Huangshui River Basin

Ruyue Yu [1], Yang Zhou [2], Shengxian Xu [1], Jing Jing [2], Hongyan Zhang [2] and Yuanfang Huang [1,*]

[1] College of Land Science and Technology, China Agricultural University, Beijing 100193, China
[2] Innovation Center of Pesticide Research, Department of Applied Chemistry, College of Science, China Agricultural University, Beijing 100193, China
* Correspondence: yfhuang@cau.edu.cn

Number of pages (including this page): 5

Number of tables: 4

Table S1. Method validation results (retention time, LOQ, calibration curves, R^2, recoveries and RSD) of HCHs and DDTs with GC–ECD.

Compounds	Retention time (min)	LOQs (μg kg^{-1})	Calibration curve (R^2)	Recovery, % (RSD, %)		
				0.005 (mg kg^{-1})	0.01 (mg kg^{-1})	0.1 (mg kg^{-1})
α-HCH	9.516	1	y = 156231002x - 370200 (0.9970)	71 (4.2)	81 (2.5)	89(3.2)
γ-HCH	10.601	1	y = 49347885x + 12455 (0.9987)	88 (5.9)	99(8.3)	96(9.8)
β-HCH	10.715	1	y = 131586543x - 232658 (0.9983)	74 (8.5)	77 (4.9)	81 (2.3)
δ-HCH	11.636	1	y = 83642218x – 148780 (0.9987)	83 (7.6)	92 (3.4)	97 (6.8)
P,P'-DDE	17.402	1	y = 101191927x - 204641 (0.9980)	92 (11.2)	98 (4.7)	96 (2.2)
O,P'-DDT	19.198	1	y = 72995934 x – 57290 (0.9999)	78 (7.4)	96 (2.4)	105 (2.7)
P,P'-DDD	19.319	1	y = 29462970x - 7246 (0.9986)	80 (7.6)	99 (5.6)	101 (6.4)
P,P'-DDT	21.083	1	y = 21590633x - 42932 (0.9939)	73 (11.0)	82 (8.2)	97 (2.3)

Table S2. Parameters required to construct a fugacity model for the Huangshui River Basin.

**Parameters**		**Value**	**Unit**
Basic physical and chemical properties of p,p'-DDE	Molar mass	3.18E+02	g/mol
	Log Kow	5.86E+00	
	Data temperature	5.50E+00	℃
	Melting point	8.90E+01	℃
	Vapor pressure	2.51E-04	Pa
	Solubility in water	4.37E-05	g/m^3
	Henry's law constant	6.31E+00	Pa m^3/mol
Half-lives of p,p'-DDE	In air	1.70E+02	h
	In aerosol	1.70E+02	h
	In water	1.70E+04	h
	In susp. particles	5.50E+04	h
	In fish	5.50E+04	h

	In soil	5.50E+04	h
	in sediment	5.50E+04	h
Volume fractions of environmental phase	Aerosol in air	4.50E-10	
	Susp. particles in water	5.54E-04	
	Fish in water	1.00E-06	
	Soil in water	2.00E-01	
	Water in soil	2.00E-01	
	Solids in soil	6.00E-01	
	Water in sediment	7.00E-01	
	Solids in sediment	3.00E-01	
Area of environmental phase	Air	2.54E+10	m^3
	Water	1.07E+08	m^3
	Soil	1.05E+10	m^3
	Sediment	1.07E+08	m^3
Depth of environmental phase	Air	6.00E+02	m
	Water	5.00E+00	m
	Soil	1.00E-01	m
	Sediment	2.00E-02	m
Advective flow residence times	Air	3.00E+01	
	Water	8.00E+02	
	Sediment	5.00E+04	
Densities for sub-compartments	Air in air	1.19E+00	kg/m^3
	Aerosol in air	1.50E+03	kg/m^3
	Water in air	1.00E+03	kg/m^3
	Susp. particles in water	2.40E+03	kg/m^3

	Fish in water	1.00E+03	kg/m^3
	Air in soil	1.19E+00	kg/m^3
	Water in soil	9.00E+02	kg/m^3
	Solid in soil	2.80E+03	kg/m^3
	Water in sediment	1.00E+03	kg/m^3
	Solid in sediment	2.26E+03	kg/m^3
Organic carbon	Susp. particles	2.60E-03	g/g
	Fish lipid	4.80E-02	g/g
	Soil	3.00E-02	g/g
	Sediment	2.00E-03	g/g
Transport velocities	Air side air–water MTC	5.00E+00	m/h
	Water side air–water MTC	5.00E-02	m/h
	Rain rate	4.55E-05	(m/h)
	Aerosol dry deposition	1.08E+01	(m/h)
	Soil–air phase diffusion MTC	2.00E-02	(m/h)
	Soil–water phase diffusion MTC	1.00E-05	(m/h)
	Soil–air boundary layer MTC	5.00E+00	(m/h)
	Sediment–water diffusion MTC	1.00E-04	(m/h)
	Sediment deposition	1.00E-06	(m/h)
	Sediment resuspension	2.00E-07	(m/h)
	Soil–water runoff rate	1.50E-05	(m/h)
	Soil–solids runoff rate	1.00E-08	(m/h)
	Scavenging ratio	2.00E+04	
Emission rate	Into air	8.31E-06	kg/h
	Into water	8.14E-04	kg/h
	Into soil	8.53E-02	kg/h

Advective inflow concentrations	Into sediment	0	kg/h
	Concentration in air	0.443	ng/m^3
	Concentration in water	154	ng/L

Table S3. SF and RfD values (children) of the compounds detected in Huangshui River Basin.

Children	C	IR	EF	ED	BW	AT	CDI	SF	RfD
α-HCH	0.75	1	350	6	30	2190	2.40E-02	6.3	5.00E-04
β-HCH	0.64	1	350	6	30	2190	2.05E-02	1.8	2.00E-04
γ-HCH	1.6	1	350	6	30	2190	5.11E-02	1.1	3.00E-04
p,p'-DDE	0.8	1	350	6	30	2190	2.56E-02	0.34	7.00E-04
p,p'-DDD	0.69	1	350	6	30	2190	2.21E-02	0.24	2.00E-03
p,p'-DDT	0.57	1	350	6	30	2190	1.82E-02	0.34	5.00E-04

Table S4. SF and RfD values (adult) of the compounds detected in Huangshui River Basin.

Adult	C	IR	EF	ED	BW	AT	CDI	SF	RfD
α-HCH	0.75	2	350	30	70	25500	8.82E-03	6.3	5.00E-04
β-HCH	0.64	2	350	30	70	25500	7.53E-03	1.8	2.00E-04
γ-HCH	1.6	2	350	30	70	25500	1.88E-02	1.1	3.00E-04
p,p'-DDE	0.8	2	350	30	70	25500	9.41E-03	0.34	7.00E-04
p,p'-DDD	0.69	2	350	30	70	25500	8.12E-03	0.24	2.00E-03
p,p'-DDT	0.57	2	350	30	70	25500	6.71E-03	0.34	5.00E-04
